# Effectiveness of text messages for decreasing inactive behaviour in patients with knee osteoarthritis: a pilot randomised controlled study

**DOI:** 10.1186/s40814-019-0494-6

**Published:** 2019-09-07

**Authors:** Cecilie Bartholdy, Henning Bliddal, Marius Henriksen

**Affiliations:** 1 The Parker Institute, Copenhagen University Hospital Bispebjerg and Frederiksberg, Copenhagen, Denmark; 2 Department of Physical and Occupational Therapy, Copenhagen University Hospital Bispebjerg and Frederiksberg, Copenhagen, Denmark

**Keywords:** Physical inactivity, Knee osteoarthritis, Accelerometer, Text messages

## Abstract

**Background:**

One of the big contributors to physical inactivity in the elderly population is osteoarthritis (OA) of the knee. Digital motivation seems to have a positive effect on individual physical inactivity level, but limited evidence exists on the effects of digital motivation on patients with knee OA.

**Objective:**

To investigate if motivational text messages reduce time spent physically inactive in patients with knee OA.

**Method:**

This study was designed as an unblinded pilot randomised controlled trial, randomising participants equally (1:1) to an intervention group (motivational text messages) or control group (no intervention). Participants were recruited from six physical therapy clinics in Denmark. Inclusion criteria were age ≥ 18, diagnosed with knee OA, owner of a smartphone or tablet, and participating or commencing participation in the GLA:D® program. The primary outcome was time spent physically inactive, measured with a tri-axial accelerometer mounted on the lateral side of the thigh. Data on OA symptoms were obtained using the Knee injury and Osteoarthritis Outcome Score (KOOS) questionnaire.

**Results:**

A total of 49 participants were screened, and 38 participants were included and randomised to either the intervention group (*n* = 19) or the control group (*n* = 19). No statistically significant difference between the two groups was found in average change of time spent physically inactive (mean difference 13.2 min/day [95% CI − 41.0 to 67.3]; *P* = 0.63), time spent standing (mean difference 3.0 min/day [95% CI − 22.7 to 28.7]; *P* = 0.81), or time spent moving (mean difference − 20.4 min/day [95% CI − 63.0 to 22.3]; *P* = 0.34) nor was there any difference in change between the two groups on KOOS.

**Conclusion:**

Motivational text messages have seemed to have no effect on overall time spent physically inactive.

**Trial registration:**

clinicaltrials.gov, NCT03339011. Registered 9 November 2017

**Electronic supplementary material:**

The online version of this article (10.1186/s40814-019-0494-6) contains supplementary material, which is available to authorized users.

## Background

Lack of physical activity and increased time spent on sedentary behaviour are major global problems as they are linked to the development of non-communicable diseases and increased risk of all-cause mortality [1–5]. One of the contributors to this trend in the elderly population is osteoarthritis (OA) of the hip or knee [6]. The current primary treatment for patients with knee OA focus on symptom relief by exercise, weight loss, pharmacological treatments, and, in advanced OA, surgery [7, 8]. These interventions have well-documented positive effects on pain, function, and quality of life [9]. However, the potential secondary effect of the initial treatments on physical activity and sedentary behaviour is not fully understood with contradicting results and limited evidence [10, 11].

Several meta-analysis and systematic reviews have established that digital motivational interventions (SMS, app, web-page, or email) can motivate to a healthy behaviour [12–14]. Studies specifically investigating change in physical activity level achieved by digital motivation in different patient groups have found an overall low to moderate effect [15–25]. However, a systematic review investigating the effect of education, exercise, or web-based interventions designed to increase the physical activity level in patients with chronic musculoskeletal pain found little to no overall change [26]. It seems that digital motivation can influence the individual’s physical activity level; however, there seems to be a limited effect for patients with chronic pain.

Exercise for patients with knee OA has a beneficial effect on their primary symptoms pain and disability [9] and can thus provide an opportunity to change physical activity or sedentary behaviour. Adding digital motivation to the period after completing an exercise intervention could therefore potentially lead to a positive change in their behaviour pattern.

Physical activity and sedentary behaviour cover a wide variety of movement intensities that are individually determined [27]. Current studies suggest that despite fulfilling the requirements for moderate-to-vigorous physical activity as suggested by guidelines, sedentary behaviour still has an adverse effect on metabolic health [28] and all-cause mortality [5]. In fact, there are indications that a reduction in sedentary behaviour is more beneficial for the health than increasing physical activity time [29]. Sedentary behaviour covers time spent sitting or reclined during wakening hours, but developments in technology now allow for assessment of movement for 24 h [30] and new terms to cover this type of outcome are needed. In this study, time spent physically inactive is used to cover the behaviours: sitting, reclined, or sleeping during 24-h recording and movement covers any other physical behaviour other than standing during 24-h recordings.

No text message-based intervention has exclusively targeted time spent physically inactive in patients with knee OA nor has such intervention been assessed in terms of efficacy on reduced risk for developing non-communicable diseases. The consequences of physical inactivity can be fatal (e.g. cardiovascular events and stroke). A clinical trial of the efficacy of text messages on risks of development of non-communicable diseases mediated through less physical inactivity would require a large study population with long-term observation under randomisation. Such a trial would be associated with research ethical problems if the effects of the intervention (text messages) on the mediator (physical inactivity) of reduced risk of non-communicable diseases are unknown. Therefore, the primary purpose of this pilot trial was to investigate the effects of motivational text messages on time spent physically inactive in patients with knee OA, who had undergone an educational and exercise intervention targeting knee OA pain and disability. It was expected that motivational text messages would decrease time spent physically inactive.

## Method

This study was designed as a pilot randomised controlled trial, randomising participants equally (1:1) to an intervention group or control group. The study was conducted from November 2017 until July 2018. The trial was registered before recruitment (www.clinicaltrials.gov: NCT03339011), and the study was deemed exempt of health research ethical approval by the Health Research Ethics Committee of the Capital Region of Denmark (record no. H-17012600) due to the nature of the trial. The trial is reported according to the CONSORT pilot and feasibility extension statement [31].

Participants were recruited from six physical therapy clinics in Denmark delivering a widely adopted educational and exercise program (the Good Life with osteoArthritis in Denmark (GLA:D®) program) [32] targeting knee OA pain and physical disability. The physical therapists introduced the trial to patients with knee OA currently participating in the educational and exercise program. Potentially eligible participants were referred to the principal investigator (CB), who screened all participants by telephone.

Inclusion criteria for this study were age ≥ 18, diagnosed with knee OA, owner of a smartphone or tablet, and participating or commencing participating in the GLA:D® program. There was one exclusion criterion: unable to read and understand Danish.

If participants were deemed eligible, a baseline visit was scheduled at the local clinic, after the last exercise session in the GLA:D® program. The follow-up visit was scheduled at the local clinic as well.

### Procedures

At the baseline visit, the participants’ demographics and questionnaire data were collected by CB. Then, physical inactivity was recorded for 3 days by a miniature accelerometer. After the 3 days of baseline data recording, participants were randomised by CB to either no intervention or text messages using a random computer-generated list that was kept in sequentially numbered, opaque, and sealed envelopes. After the 6-week intervention period, questionnaire data was collected and additionally 3 days of accelerometer recording was collected.

### Motivational text message intervention

The participants randomised to the intervention group received three text messages per week on their mobile phone during the 6-week intervention period, totalling 18 messages (two messages during the weekdays and one during the weekends). The text messages were sent anonymously, and replies were not possible.

The text messages contained information and general advice about the importance of performing daily physical activity. The intervention was based on previous experience with web-based digital motivation for hip and knee OA [33], and the proven effectiveness of weekly text messages for decreasing sedentary behaviour [17, 21]. The content of the text messages was developed based on recommendation and advice from the Danish Health Authority about the importance of regular daily physical activity. The original Danish version and an English translation of the phrases are presented in Additional file 1.

### Control group

Participants allocated to the control group received no attention from the study during the intervention period.

### Outcomes

#### Physical inactivity measurement

The SENS motion® system was used to record physical inactivity. The SENS motion® system consists of a single-use miniature tri-axial accelerometer (dimensions 50 × 21 × 5 mm, weight 8 g; SENS-MOTION® activity measurement system, version 1.7.1) and a dedicated smartphone application. The system measures movement continuously at 12.5 Hz (every 10 s), 24 h a day, and have a battery lifespan of approximately 20 weeks. The accelerometer was placed on the lateral side of the thigh at the baseline visit with a small waterproof Band-Aid (Medipore™, 3M, Soft Cloth Surgical Tape on Liner) and was worn continuously during the observation period (from baseline to follow-up). The accelerometer has an onboard memory of 14 days and was connected to an app downloaded to the participants’ smartphones. Data from the accelerometer was uploaded to the app via Bluetooth and then transmitted to a secured web server for storage and subsequent analysis. Participants were not provided with feedback on physical inactivity. To avoid loss of data (due to full memory), participants had to connect the accelerometer to the app at least once weekly. The discretely worn accelerometer does not interfere with the participant’s normal life [30]. The accelerometer is waterproof, and it is therefore not necessary to remove it during bathing, swimming, and showering.

During the study period, participants could change the Band-Aid if needed and replace the accelerometer on the opposite thigh if necessary. This has previously been shown to be of no consequence for the measurements [30]. An instruction sheet and additional Band-Aids were provided at the baseline visit.

The system has a built-in algorithm that categorised data into inactivity (sitting, reclined, or sleeping), standing, walking, cycling, and other activities. Based on a previous study assessing the validity of data in knee OA patient [30] and previous literature [34], data was divided into three categories: time spent physically inactive (sitting, reclined, and sleeping), time spend standing, and time spend moving (e.g. walking, running, cycling, and other activities).

These three categories were summed and averaged as minutes per day over the three baseline and follow-up days, respectively. In this study, the main outcome was time spent physically inactive (minutes/day).

#### Patient-reported knee symptoms

The participants’ knee symptom severity on pain, disability, and quality of life was assessed at the baseline and follow-up visits using the Knee injury and Osteoarthritis Outcome Score (KOOS) questionnaire [35]. The KOOS is considered a reliable and valid tool to assess both short- and long-term changes in patients’ opinion about their knee problems [35–37]. The questionnaire consists of 42 items divided over five subscales: KOOS pain, KOOS symptoms, KOOS ADL (function in daily living), KOOS sport/rec (function in sport and recreation), and KOOS QOL (knee-related quality of life). Answers were given on a 5-point Likert scales scoring from 0 to 4. A standardised score was calculated (0–100) with 100 indicating no symptoms and 0 indicating extreme symptoms. A change between 8 and 12 points was considered clinically relevant.

#### Self-reported change in physical inactivity

To assess if participants felt that they had changed their amount of time spent physically active, they were asked: “Have you changed the amount of time you spent physical activity from before you entered this study until know”. The participants were given nine possible answers:

- I have spent less time on physical activity
- No change in time spent physically active
- ½–1 h more physical activity per week
- 1–1½ h more physical activity per week
- 1½–2 h more physical activity per week
- 2–2½ h more physical activity per week
- 2½–3 h more physical activity per week
- 3–3½ h more physical activity per week
- More than 3½ h more physical activity per week

### Statistical methods

The analyses were performed on the intention to treat (ITT) population meaning all that completed the baseline visit. The main outcome was change from baseline between groups in time spent physically inactive (minutes). Baseline was defined at the first 3 days of measurement and follow-up was defined as the last 3 days of measurement obtained before and after the 6-week intervention period. Analysis of covariance (ANCOVA) adjusted for baseline values was performed to assess difference between the two groups in the average change in time spent physically inactive, time spent moving, time spent standing, and KOOS.

In addition, sensitivity analyses were conducted further adjusting for age, baseline KOOS function, baseline KOOS pain, and baseline KOOS symptoms. All available data was used. For missing data, last observation was carried forward.

The sample size was pragmatically set to 50 participants (25 in each group). All analyses were performed using commercially available statistical software (SAS, version 9.4; SAS Institute Inc).

## Results

**Fig. 1:**
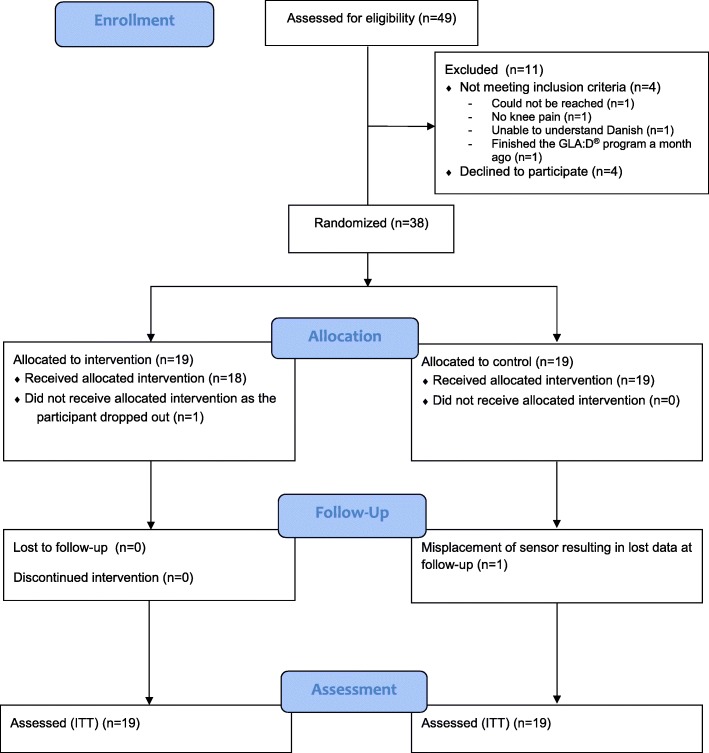
Flow of participants throughout the study

Due to funding restriction, recruitment was stopped before reaching the planned sample size of 50. A total of 38 participants were included in the study; of these, two had missing data at the follow-up visit: one withdrew from participation and was therefore ruled as drop-out, the other lost the accelerometer resulting in lost data at the follow-up days. Baseline characteristics for all 38 participants are presented in Table 1. The flow of participants is presented in Fig. 1.

**Table 1:** Baseline characteristics of all participants, participants in the intervention group and in the control group, presented as mean and standard deviation (SD)

	Intervention (*N* = 19)	Control (*N* = 19)
Gender, no. (%)		
Female	15 (79.0)	14 (73.7)
Male	4 (21.1)	5 (26.3)
Affected knee, no. (%)		
Left	12 (63.2)	6 (31.6)
Right	7 (36.8)	13 (68.4)
Age (years)	68 (7.3)	62 (9.7)
Body weight (kg)	80.9 (16.1)	80.8 (13.2)
Height (cm)	168.7 (8.3)	170.3 (8.0)
BMI (kg/m^2^)	28.3 (4.5)	27.9 (4.0)
Physical activity measures		
Inactivity (min/day)	1051.5 (98.7)	1048.5 (98.5)
Standing (min/day)	131.0 (51.9)	118.5 (55.5)
Movement (min/day)	257.5 (67.6)	273.1 (66.6)
KOOS (0–100)		
Function (ADL)	70.5 (15.4)	80.9 (14.3)
Quality of life (QoL)	48.0 (17.1)	52.0 (15.9)
Pain	62.4 (15.9)	76.2 (13.4)
Sport/rec	35.5 (22.5)	39.2 (26.6)
Symptoms	64.7 (19.6)	75.8 (15.5)

*Abbreviations*: *KOOS* Knee injury and Osteoarthritis Outcome Score, where 0 is worst and 100 indicates no symptoms; *Activity* sum of walking, other, exercise, and cycling; *BMI* body mass index

### Physical inactivity and activity measures

There were no meaningful differences between groups in average change in time spent physically inactive (mean difference 13.2 min [95% CI − 41.0 to 67.3]; *P* = 0.63), time spent standing (mean difference 3.0 min [95% CI − 22.7 to 28.7]; *P* = 0.81), or time spent moving (mean difference − 20.4 min [95% CI − 63.0 to 22.3]; *P* = 0.34) nor were there any differences between the groups in the changes in the KOOS (Table 2). Sensitivity analysis did not change the outcome (Additional file 2).

**Table 2:** Change scores for each group with standard error (SE). Difference in change between the intervention and control groups is presented as mean with 95% CI and *p* value

Change in	Intervention (*N* = 19)	Control (*N* = 19)	Mean difference in change between groups	
	Mean change (SD) [SE]	Mean change (SE)	Mean (95% CI)	*P* value
Time spent inactive (min/day)	5.5 (82.2) [18.9]	− 7.6 (82.2) [18.9]	13.2 (− 41.0 to 67.3)	0.63
Time spent standing (min/day)	− 2.1 (38.9) [8.9]	− 5.0 (38.9) [8.9]	3.0 (− 22.7 to 28.7)	0.81
Time spent moving (min/day)	− 5.6 (64.6) [14.8]	14.8 (64.6) [14.8]	− 20.4 (− 63.0 to 22.3)	0.34
KOOS				
Function	1.8 (11.3) [2.6]	4.4 (11.3) [2.6]	− 2.6 (− 10.2 to 5.1)	0.50
Quality of life	3.4 (14.6) [3.3]	3.1 (14.6) [3.3]	0.32 (− 9.3 to 9.9)	0.95
Pain	2.6 (15.5) [3.6]	5.0 (15.5) [3.6]	− 2.5 (− 13.2 to 8.2)	0.64
Sport/rec	7.9 (18.9) [4.3]	8.6 (18.9) [4.3]	− 0.69 (− 13.2 to 11.8)	0.91
Symptoms	2.5 (12.4) [2.8]	4.2 (12.4) [2.8]	− 1.7 (− 10.1 to 6.7)	0.68

*Abbreviations*: *KOOS* Knee injury and Osteoarthritis Outcome Score, where 0 is worst and 100 indicates no symptoms; *Activity* sum of walking, other, exercise, and cycling; *BMI* body mass index

### Self-reported change in physical activity

**Fig. 2:**
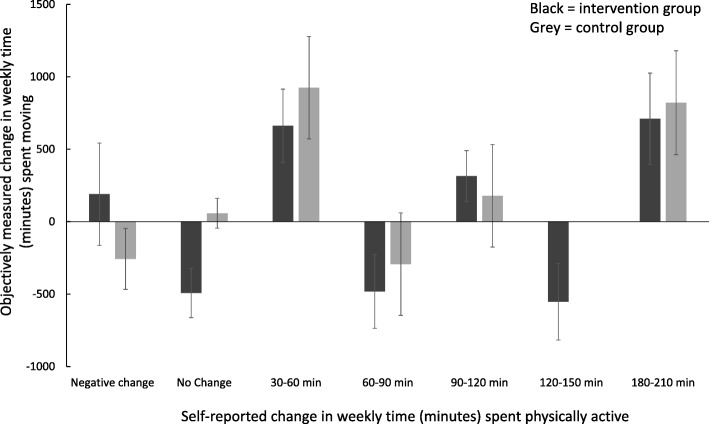
Graphical illustration of the relationship between responses on self-reported change in weekly time (minutes) spent physically active (horizontal axis) and the average (error bars: standard error) objectively measured change in weekly time (minutes) spent moving. Error bars indicate standard errors of the mean, and positive values on the *y*-axes indicate an increase in time spent moving. The black columns represent the intervention group, and the grey columns represent the control group

The distribution of self-reported change inactivity in the two groups is presented in Table 3. Twelve (66%) participants in the intervention group reported a positive change in physical activity, whereas only four in the control group reported such a positive change. Figure 2 illustrates the relationship between self-reported weekly change in physical activity (horizontal axis) and objectively measured weekly changes in time spent moving for each group (vertical axis). The figure indicates a lack of coherence between objectively measured changes in movement and self-reported change in time spent physically active.

**Table 3:** Distribution of the answers to the self-reported changes in physical activity questionnaire for each group

Self-reported change in physical activity	Intervention (*n* = 18*)	Control (*n* = 19)
Reduction in physical activity, no. (%)	1 (2.7)	3 (8.1)
No change in physical activity, no. (%)	5 (13.5)	12 (32.4)
Weekly increase of ½–1 h, no. (%)	2 (5.4)	1 (2.7)
Weekly increase of 1–1½ h, no. (%)	2 (5.4)	1 (2.7)
Weekly increase of 1½–2 h, no. (%)	4 (10.8)	1 (2.7)
Weekly increase of 2–2½ h, no. (%)	2 (5.4)	0 (0)
Weekly increase of 3–3½ h, no. (%)	2 (5.4)	1 (2.7)

*One participant dropped out before the follow-up visit

## Discussion

In this pilot study, a low-tech low-cost intervention was applied immediately after completing a well-established education and exercise program to assess if, with a small effort, time spent physically inactive could be reduced.

Overall, no difference in time spent physically inactive, time spent standing, or time spent moving was found. While this pilot study is small, the results do not indicate any potentially beneficial effects as judged by the precision of the estimates (95% CI) neither was there a meaningful difference between the two groups in the KOOS. This suggests that motivational text messages have no meaningful effect on time spent physically inactive in knee OA patients in the period after having completed an education and exercise program. Hence, text messages with general information and advice about physical activity do not seem to be a solution to decrease physical inactivity and thereby improve health in this population.

This pilot study aimed to assess the short-term effect of the low-tech low-cost intervention on physical inactivity. A positive effect would have pointed at a cheap and simple way of supporting activity and reducing inactivity and inform a larger trial on the efficacy on risk for developing inactivity related diseases.

The intervention type could explain the lack of change. Without educational sessions or individual consultations explicitly focusing on how to reduce time spent physically inactive, participants only had the motivational text messages to help them. Other studies aiming at increasing physical activity level have used educational sessions with small effects in short term [10]. The relatively small effect from other trials and no effect in this trial could indicate that the motivational text messages are too simple and additional attention is needed to change the behaviour in this patient group. However, in the educational and exercise program participants completed before inclusion in this study, they received education about the importance of moving and how to move with limited pain [32]. Despite this, the results of this study suggest that that the impact of the intervention was too low to facilitate a change in physically inactive behaviour.

The intervention consisted of motivational text messages based on the Danish Health Authority statement about the importance of regular daily physical activity. The focus in the motivational text messages was to increase time spent physically active and thereby reduce time spent physically inactive. The content of the text messages could have focused more on the importance of moving in general instead of addressing specific types of activities which might have been more effective [29].

A study targeting reduction in total sitting time in patients with rheumatoid arthritis using three motivational sessions and text message reminders found a reduction in total sedentary time [38]. Another study investigating the effects of different treatments after a weight loss intervention found that in rather sedentary subjects with knee OA, the offer of an activity program had low adherence [39]. These results combined with our study could indicate that an intervention aiming at reducing time spent physically inactive needs to be specific and patient tailored to achieve an effect. Furthermore, standing on a specific behaviour change theory in the development of a behaviour change intervention seems to increase the chance of success [40–42].

For every 1-h increase in sitting time, the hazard ratio for all-cause mortality increases by 1.05 (95% CI 1.02–1.08) [4]. In this study, the baseline average time spent physically inactive was 17.5 h per day, of which approximately 8 h would be sleep. This leaves approximately 9.5 h of sedentary time (sitting or reclined) during the day. This suggests that a 1-h reduction in sedentary time per day in this patient group would indeed be possible. However, we did not reach a reduction. In fact, a reduction was observed neither within nor between groups (Table 2).

Self-reported change in weekly physical activity and objective measured movement did not seem to be related in any of the two groups (Fig. 2). Our results indicate that the participants’ experience of their changes in physical activity tends to give unreliable and often overestimated changes in physical activity behaviour [43], and our results support the use of accelerometers to give more reliable information.

The primary outcome was time spent physically inactive as it has proven both easy to measure [30] and a reduction of total physical inactivity time is desirable to reduce all-cause mortality [4]. It is possible that the intensity of the participants’ movement had changed and thereby provided them with additional health benefits [44]. However, despite potential change in physical activity intensities, a lack of reduction in physical inactivity still affect the health negatively [5].

A slight imbalance was present between the intervention group and the control group with the intervention group being slightly older, less active, and with worse symptoms. However, no changes within each group in any outcome (Table 2) were observed suggesting that the difference had no influence over the results.

An important limitation is the number of participants, but due to funding restrictions, this pilot study was cut short. However, based on the precision of our results, it is unlikely that a larger number of participants would have changed the outcome significantly. Another limitation to this pilot study is the lack of evaluation of trial procedures as part of assessing feasibility of this pilot study. A framework for development and evaluation of complex interventions has been developed to assess relevant element of such an intervention [45]. The use of such framework to develop this intervention might have improved the quality of the intervention. Furthermore, using a mixed-method process evaluation to assess multiple aspects of the trial such as recruitment rate, dosage, and intervention fidelity would have added valuable information. However, the efficacy results suggest that focus needs to be on the content and contextual factors of the intervention rather than on feasibility of the study procedures.

## Conclusion

In this pilot study, motivational text messages about physical activity did not reduce overall time spent physically inactive in patients with knee OA who prior to the study had completed an education and exercise program. Thus, a larger trial assessing this intervention is not warranted. Instead, other methods for reinforcing reduced time spent physically inactive are needed in patients with knee OA.

## Additional files

Additional file 1: Motivational text messages. (DOCX 17 kb)

Additional file 2: Sensitivity analysis. (DOCX 17 kb)

## Data Availability

No additional data available.

## Abbreviations

GLA:D®: The Good Life with osteoArthritis in Denmark program
ITT: Intention to treat
KOOS: Knee injury and Osteoarthritis Outcome Score
OA: Osteoarthritis
