# Supplementary material for: Effectiveness of text messages for decreasing inactive behaviour in patients with knee osteoarthritis: a pilot randomised controlled study
Source: Pilot Feasibility Stud. 2019 Sep 7;5:112. doi: 10.1186/s40814-019-0494-6 (PMC6732192; doi:10.1186/s40814-019-0494-6)
Supplement: Supplementary file 1 — Motivational text messages. (DOCX 17 kb) [file 40814_2019_494_MOESM1_ESM.docx]

**MOTIVERENDE TEKST BESKEDER**

1. Sæt farten op på din gåtur og mærk din puls stige. Det er god motion.
2. Husk at fysisk aktivitet er meget sundt for dig. Det nedsætter risikoen for mange ting fx sukkersyge og hjertesygdom.
3. Det anbefales at være fysisk aktiv mindst 30 minutter hver dag.
4. Tag trappen HVER GANG du møder en. Det giver både styrke og udholdenhed.
5. Regelmæssig fysisk aktivitet kan sænke blodtrykket – det er sundt.
6. Regelmæssig fysisk aktivitet og træning kan bedre helbredet.
7. Træning og fysisk aktivitet er godt for både fysisk og psykisk velvære. Og så kan det forbedre dit humør.
8. Har du trænet eller været fysisk aktiv I dag?
9. En gåtur I raskt tempo er god fysisk aktivitet. Gør det sammen med en ven – så bliver det også hyggeligt.
10. En gåtur er gratis og kan gøres overalt. Husk at tage gode sko på og tøj der passer til vejret.
11. Prøv at gøre gåture til en del af din hverdag. Parker langt væk fra butikkerne når du skal handle.
12. Prøv at være mere fysisk aktiv i din hverdag. Gå når du skal handle, eller tag en tur i skoven.
13. Husk at være fysisk aktiv mindst 30 minutter om dagen. Du må gerne dele det op i 3 perioder a 10 minutter.
14. Der er mange måder at være fysisk aktiv på. Prøv fx Tai Chi, Pilates, svømning, gåture, havearbejde, yoga eller dans.
15. En daglig hurtig gåtur er godt for dig.
16. Fysisk aktivitet giver dig mere energi i hverdagen.
17. En daglig cykeltur er godt for dig.
18. Prøv at tage cyklen i stedet for bilen når du skal besøge dine venner.

**MOTIVATIONAL TEXT MESSAGES(TRANSLATED)**

1. Increase your speed when walking and feel the heartrate increase. That is good exercise
2. Physical activity is healthy. It reduces the risk of developing disease e.g. diabetes or heart diseases.
3. It is recommended that you spent a minimum of 30 minutes on physical activity each day.
4. Take the stairs every time. It builds strength and gives endurance.
5. Regular physical activity gives a healthy blood pressure.
6. Regular physical activity and exercise gives a better health
7. Exercise and physical activity is beneficial both for mental and physical health. It can improve your mood.
8. Have you exercised or been physically active today?
9. A daily walk in high speed is good for your health. Ask a friend to join.
10. Walking is free and can be done anywhere. Remember to dress for the weather.
11. Try to make walk part of your everyday routine. Park fare away when you go shopping.
12. Try to increase your daily physical activity level. Walk down to the grocery store or take a walk in the forest.
13. Remember to be physically active minimum 30 minutes a day. It is okay to divided it in three periods of 10 minutes each.
14. You can be physically active in many ways. Try Thai Chi, Pilates, swimming, walking, gardening, yoga or dancing.
15. A daily walk is good for your health.
16. Daily physical activity will give you more energy.
17. A daily bike strip is good for your health.
18. Try taking the bike in steed of the car when you need to visit friends.
