# Supplementary material for: Effectiveness of text messages for decreasing inactive behaviour in patients with knee osteoarthritis: a pilot randomised controlled study
Source: Pilot Feasibility Stud. 2019 Sep 7;5:112. doi: 10.1186/s40814-019-0494-6 (PMC6732192; doi:10.1186/s40814-019-0494-6)
Supplement: Supplementary file 2 — Sensitivity analysis. (DOCX 17 kb) [file 40814_2019_494_MOESM2_ESM.docx]

|  | **Intervention**  **(n=19)** | | **Control**  **(N=19)** | **Mean difference in change between groups** | | | |
| --- | --- | --- | --- | --- | --- | --- | --- |
| **Outcomes** | *Mean change*  *(SE)* | *Mean change*  *(SE)* | | *Mean*  *(95% CI)* | *P-value* | |  |
| Time spent inactive (min/day) | 4.4  (21.2) | -6.5  (21.2) | | 10.9  (-55.6 to 77.5) | 0.74 | |  |
| Time spent standing (min/day) | -0.70  (9.8) | -6.3  (9.8) | | 5.6  (-25.0 to 36.3) | 0.71 | |  |
| Time spent moving (min/day) | -5.7  (16.4) | 14.9  (16.4) | | -20.6  (-72.1 to 30.9) | 0.42 | |  |
| ***KOOS*** |  |  | |  |  | |  |
| Function | 0.27  (2.7) | 5.8  (2.7) | | -5.6  (-14.0 to 2.8) | 0.19 | |  |
| Quality of Life | 1.9  (3.6) | 4.7  (3.6) | | -2.8  (-14.2 to 8.5) | 0.61 | |  |
| Pain | 1.7  (3.4) | 6.0  (3.4) | | -4.3  (-14.9 to 6.3) | 0.41 | |  |
| Sport/rec | 4.6  (4.6) | 12.0  (4.6) | | -7.3  (-21.7 to 7.1) | 0.31 | |  |
| Symptoms | 0.13  (2.8) | 6.6  (2.8) | | -6.5  (-15.1 to 2.1) | 0.13 | |  |
| ***Abbreviation***: KOOS, knee Injury and Osteoarthritis Outcome Score, where 0 is worst and 100 indicates no symptoms; Activity, sum of walking, other, exercise, and cycling; BMI, body mass index.  *Significant change from baseline | | | | | |  |  |

**Sensitivity analysis:** Difference in change between the intervention and control group adjusted for age, KOOS function, KOOS pain, and KOOS symptoms. Presented as mean with 95% confidence interval (CI) and p-value.
